# Economics Risks of and Growth of the High Deductible Health Plan Among Patients With Cognitive Impairment

**DOI:** 10.1093/geroni/igaa057.338

**Published:** 2020-12-16

**Authors:** Kevin Lu, Sam Li, Jing Yuan, Minghui Li

**Affiliations:** 1 University of South Carolina, Columbia, South Carolina, United States; 2 University of Tennessee Health Science Center, Memphis, Tennessee, United States; 3 Rutgers University, Piscataway, New Jersey, United States

## Abstract

OBJECTIVES: High Deductible Health Plan (HDHP) is characterized by higher deductibles and lower monthly premiums. Nevertheless, health economists are concerned that HDHPs may reduce or delay needed care, which will ultimately lead to poorer access to care for chronically affected participants. The objectives of this research are 1) to investigate the HDHP enrollment trend over the past decade; and, 2) to determine the effects of HDHP on risks of financial access risks among adults with cognitive impairment (CI). METHODS: Data were obtained between 2010-2018 from National Health Interview Survey (NHIS). Financial access to healthcare was assessed based on 6 survey questions by CDC. For data analysis, simple T tests and Chisq tests were used where appropriate, with multi-variable logistic regressions implemented to evaluate the effects of HDHP on risks of financial access. RESULTS: Of the 103,649 enrollments, 1,148 were with cognitive impairment and 102,501 were without CI diagnosis. A 55% increase in HDHP registers with cognitive impairment was observed from 2010 (30.50%) to 2018 (47.24%). After controlling for confounding variables, patients with HDPHs were more likely to have risks of financial access compared to those without HDHP (OR= 1.313, 95% CI, 1.002-1.719, p=0.0483). CONCLUSIONS: HDHPs are intended to support effective care options and reduce health care costs. Our research among CI patients with HDHP experienced more financial access risks than those without HDHP, indicating that HDHPs might have unintended consequences of healthcare usage. Employers and health care decision-makers may need to consider providing compensation to those HDHP enrollers with CI.

